# Supplementary material for: Synthetic Analogues of Aminoadamantane as Influenza Viral Inhibitors—In Vitro, In Silico and QSAR Studies
Source: Molecules. 2020 Sep 1;25(17):3989. doi: 10.3390/molecules25173989 (PMC7504818; doi:10.3390/molecules25173989)
Supplement: Supplementary file 1 [file molecules-25-03989-s001.pdf]

Supplementary information file for the article

Synthetic Analogues of Aminoadamantane as Influenza Viral Inhibitors—In Vitro, In Silico and QSAR Studies

Radoslav Chayrov [1], Nikolaos A. Parisis [2], Maria V. Chatziathanasiadou [2], Eleni Vrontaki [3,4], Kalliopi Moschovou [3], Georgia Melagraki [5], Hristina Sbirkova-Dimitrova [6], Boris Shivachev [6], Michaela Schmidtke [7], Yavor Mitrev [8], Martin Sticha [9], Thomas Mavromoustakos [3], Andreas G. Tzakos [2,*] and Ivanka Stankova [1,*]

[1] Department of Chemistry, South-West University "Neofit Rilski", Blagoevgrad, 2700, Bulgaria; rchayrov@swu.bg
[2] Section of Organic Chemistry and Biochemistry, Department of Chemistry, University of Ioannina, 45110 Ioannina, Greece; nikparis@gmail.com (N.A.P.); m.chatziathanasiadou@gmail.com (M.V.C.)
[3] Division of Organic Chemistry, Department of Chemistry, National and Kapodistri`an University of Athens, 15771 Zografou, Greece; kmoschovou@chem.uoa.gr (K.M.); tmavrom@chem.uoa.gr (T.M.); evrontaki@pharm.uoa.gr (E.V.)
[4] Division of Pharmaceutical Chemistry, Department of Pharmacy, National and Kapodistrian University of Athens, 15771 Zografou, Greece
[5] Division of Physical Sciences and Applications, Department of Military Sciences, Hellenic Military Academy, Vari-Koropi Avenue, 16672 Vári, Greece; georgiamelagraki@gmail.com
[6] Institute of Mineralogy and Crystallography "Acad. Ivan Kostov", Bulgarian Academy of Sciences, 1113 Sofia, Bulgaria; sbirkova@mail.bg (H.S.-D.); bls@clmc.bas.bg (B.S.)
[7] Friedrich Schiller University, Department of Virology and Antiviral Therapy, 207745 Jena, Germany; michaela.schmidtke@med.uni-jena.de
[8] Institute of Organic Chemistry with centre of Phytochemistry, Bulgarian Academy of Science, 1113 Sofia, Bulgaria; yavor@orgchm.bas.bg
[9] Department of Analytical Chemistry, Charles University, 11636 Prague1, Czech Republic; martin.sticha@natur.cuni.cz
* Correspondence: atzakos@uoi.gr (A.G.T.); ivastankova@abv.bg (I.S.); Tel/Fax: +359-73-88-55-16

Figure S1. 1D ^{1}H NMR spectrum Alanyl-rimantadine 3
Figure S2. 1D ^{13}C NMR spectra Alanyl-rimantadine 4
Figure S3. 1D ^{1}H NMR spectrum Glycyl-rimantadine 5
Figure S4. 1D ^{13}C NMR spectra Glycyl-rimantadine 6
Figure S5. 1D ^{1}H NMR spectrum Isoleucyl-rimantadine 7
Figure S6. 1D ^{13}C NMR spectra Isoleucyl-rimantadine 8
Figure S7. 1D ^{1}H NMR spectrum Phenylalanyl-rimantadine 9
Figure S8. 1D ^{13}C NMR spectra Phenylalanyl-rimantadine 12
Figure S9. 1D ^{1}H NMR spectrum D-(4-F)-phenylalanyl-rimantadine 11
Figure S10. 1D ^{13}C NMR spectra D-(4-F)-phenylalanyl-rimantadine 12
Figure S11. 1D ^{1}H NMR spectrum L-(4-F)-Phenylalanyl-rimantadine 13
Figure S12. 1D ^{13}C NMR spectrum L-(4-F)-Phenylalanyl-rimantadine 15
Figure S13. 1D ^{13}C NMR spectra Valyl-rimantadine 16

Figure S14. 1D ^{13}C NMR spectra Valyl-rimantadine 16
Figure S15. 1D ^{1}N NMR spectra β-Alanyl -rimantadine 17
Figure S16. 1D ^{1}H NMR spectrum Tyrosinyl-rimantadine 18
Figure S17. 1D ^{13}C NMR spectra Tyrosinyl-rimantadine 19
Figure S18. 1D ^{1}H NMR spectrum Alanyl-amantadine 20
Figure S19. 1D ^{13}C NMR spectra Alanyl-amantadine 23
Figure S20. 1D ^{1}H NMR spectrum Phenylalanyl-amantadine 22
Figure S21. 1D ^{13}C NMR spectra Phenylalanyl-amantadine 23
Figure S22. 1D ^{1}H NMR spectrum (4-F)-Phenylalanyl-amantadine 24
Figure S23. 1D ^{13}C NMR spectrum (4-F)-Phenylalanyl-amantadine 24
Figure S24. 1D ^{1}H NMR spectrum Valyl-amantadine. 26
Figure S25. 1D ^{13}C NMR spectra Valyl-amantadine. 27
Table S1. Crystallographic data-collection statistics for glycyl-rimantadine. 28
Table S2. Selected geometric parameters for Glycyl-rimantadine. Bond Lengths [Å] and Bond Angles [°]. 28

Alanyl-rimantadine (4a). **^{1}H-NMR:** ($CDCl_3$) δ (ppm): 1.02. (d, J=5.7 Hz, 3H, H-2′, D1), 1.00. (d, J=5.9 Hz, 3H, H-2′, D2), 1.34. (d, J=6.8 Hz, 3H, CH_3-Ala, D1), 1.37. (d, J=6.8 Hz, 3H, CH_3-Ala, D2), 1.453 (s, 9H, tBu, D1+D2), 1.465 (m, 3H, H-2a*, D1+D2), 1.532 (m, 3H, H-2b*, D1+D2), 1.611 (m, 3H, H-4b, D1+D2), 1.698 (m, 3H, H-4a, D1+D2), 1.975 (br t, 3H, H-3, D1+D2), 3.667 (br, 3H, H-3′,D1+D2), 4.110 (br, 1H, CH-Ala, D1+D2), 4.99 (br, 1H, NH), 6.116 (br, 1H, NH, D1), 6.07(br, 1H, NH, D2); **^{13}C-NMR:** ($CDCl_3$) δ (ppm): 14.5 (CH_3, C-2′, D1+D2), 17.6 (CH_3-Ala, D1), 18.3 (CH_3-Ala, D2), 28.2 (CH_3, C-3*), 28.3 ($C(CH_3)_3$*), 35.7 (C-1′), 36.9 (CH_2, CH_2,C-4, D1+D2), 38.3 (CH_2), 44.9 (CH_2), 53.1 (CH), 80.3 (Cq, tBu), 155.6 (CO-Boc), 171.9 (CO); **ESI-MS:** 389 $[M+K]^+$, 373 $[M+Na]^+$, 351 $[M+H]^+$; m.p.=157-159 °C; **yield=74%.**

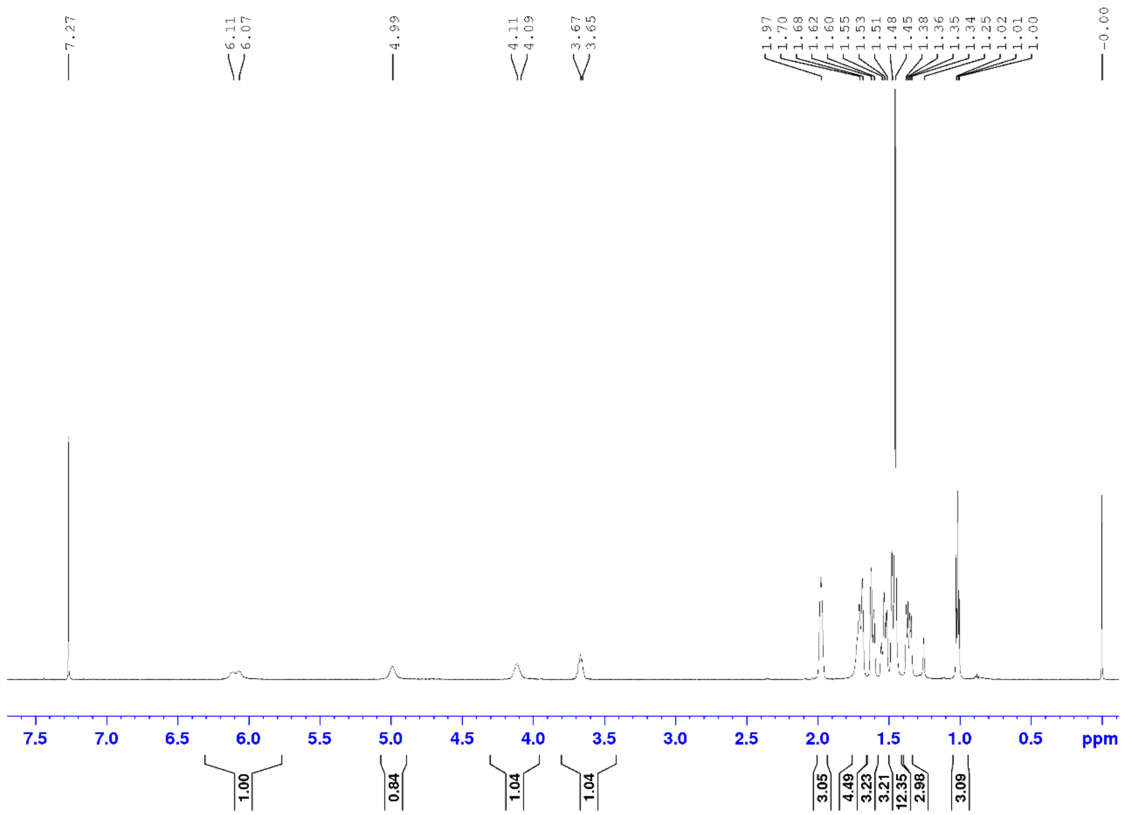

Figure S1. **1D ^{1}H NMR spectrum Alanyl-rimantadine**

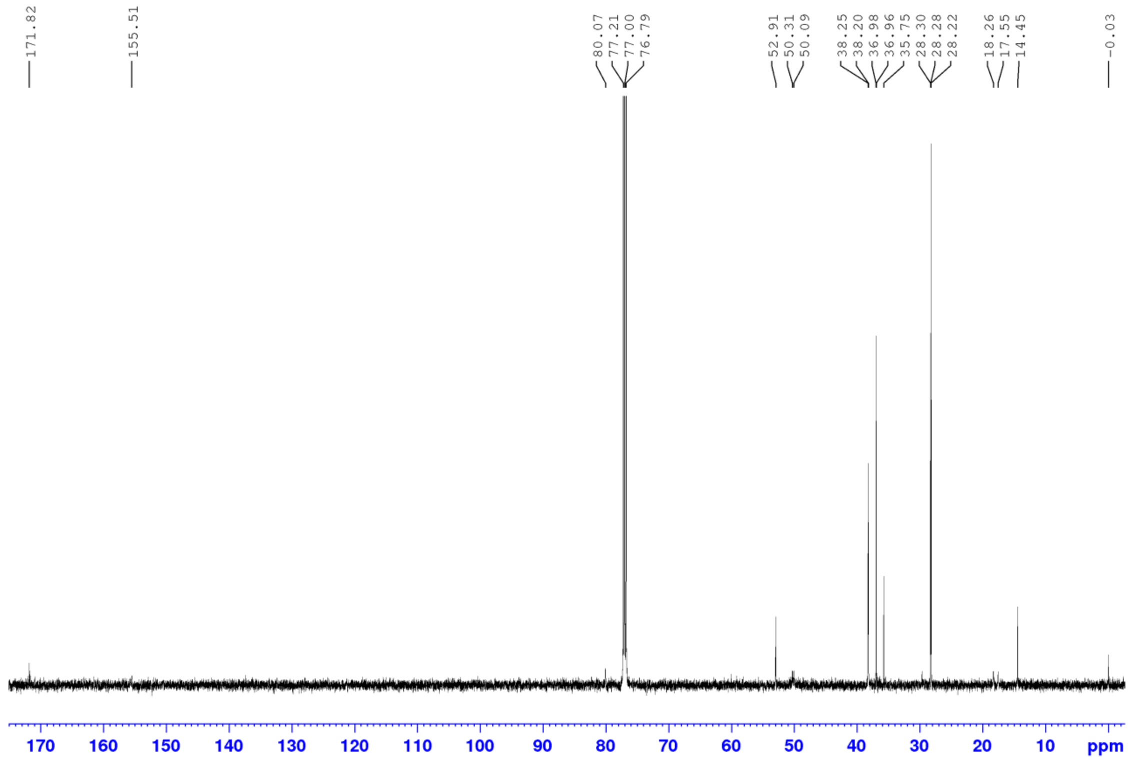

Figure S2. **1D ^{13}C NMR spectra Alanyl-rimantadine**

Glycyl-rimantadine (4b). ^{1}H-NMR: ($CDCl_3$) δ (ppm): 1.028. (d, J=6.8 Hz, 3H, H-2′), 1.466 (s, 9H, tBu), 1.462 (m, 3H, H-2a*), 1.538 (m, 3H, H-2b*), 1.613 (m, 3H, H-4b), 1.700 (m, 3H, H-4a), 1.981 (br t, 3H, H-3), 3.69 (br t, 1H, H-3′), 5.16 (br, 1H, NH), 6.07 (br, 1H, NH); **^{13}C-NMR:** ($CDCl_3$) δ (ppm): 14.5 (CH_3, C-2′), 28.2 (CH_3, C-3*), 28.3 ($C(CH_3)3$*), 35.7 (C-1′), 36.9 (CH_2,C-4), 38.3 (CH_2, C-2), 44.9 (CH_2), 53.1 (CH, C-3′), 80.3 (Cq, tBu), 156.2 (CO-Boc), 168.8 (CO); **ESI-MS:** 695 $[2M+Na]^+$, 359 $[M+Na]^+$; m.p.=165-169 °C; **yield = 77%.**

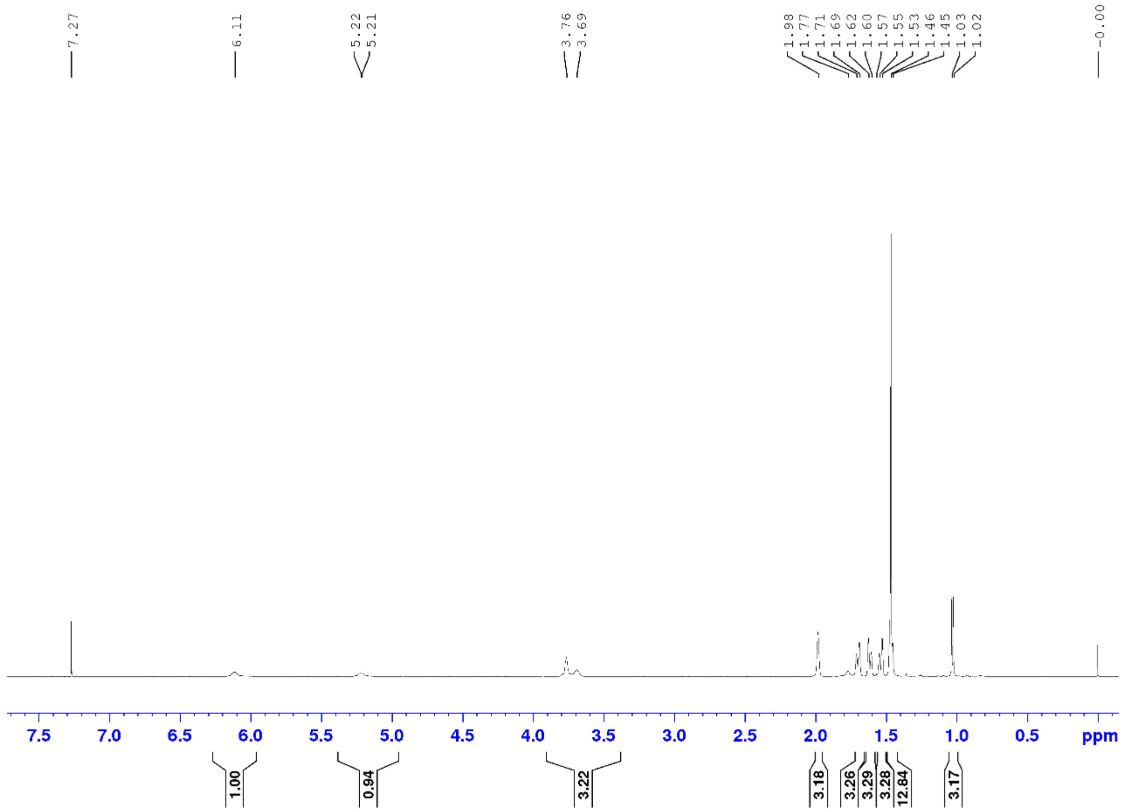

Figure S3. 1D ^{1}H NMR spectrum Glycyl-rimantadine

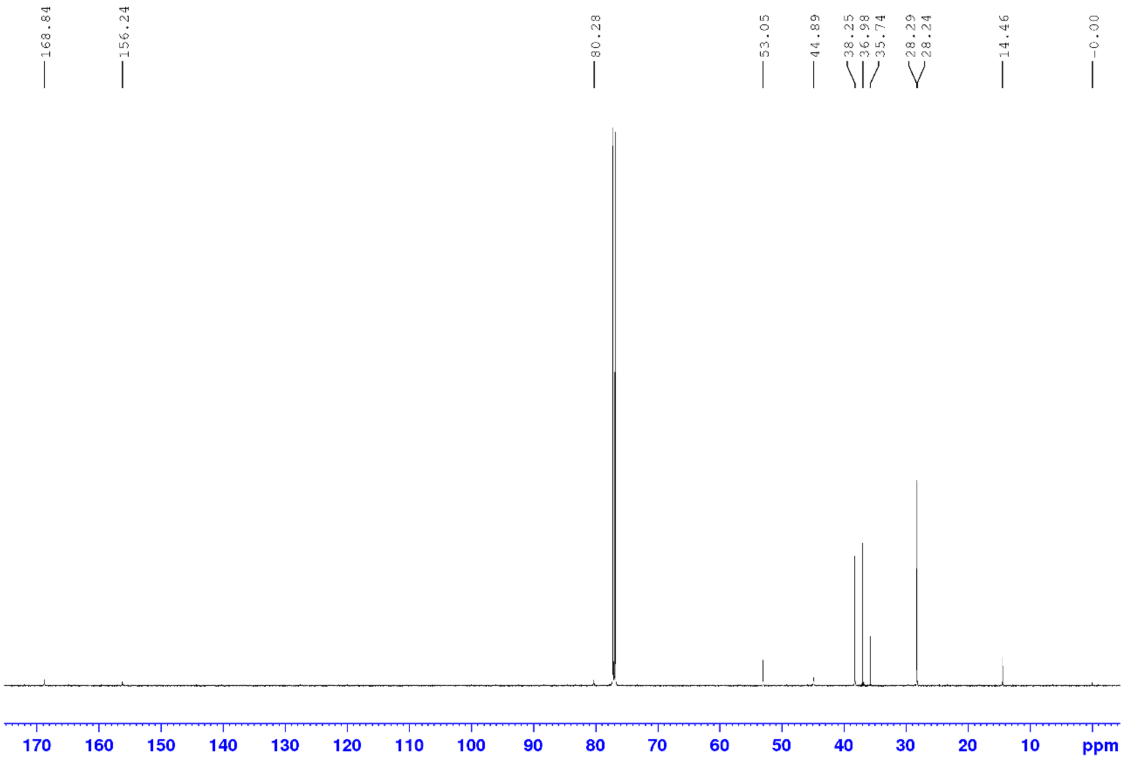

Figure S4. 1D ^{13}C NMR spectra Glycyl-rimantadine

Isoleucyl-rimantadine (4c). ^{1}H-NMR: ($CDCl_3$) δ (ppm): 0.91. (3H, Ile, D), 0.92. (3H, Ile, D) , 0.96. (3H, Ile, D)1.01. (d, J=6.7 Hz, 3H, H-2′, D1), 1.02 (d, J=6.7 Hz, 3H, H-2′, D2), 1.13 (m, 1H, CH_2-Ile, D1+D2), 1.53 (m, 1H, CH_2-Ile, D1+D2), 1.43 (s, 9H, tBu, D1), 1.44 (s, 9H, tBu, D2), 1.465(m, 3H, H-2a*, D1+D2), 1.54 (m, 3H, H-2b*, D1+D2), 1.61 (m, 3H, H-4b, D1+D2), 1.70 (m, 3H, H-4a, D1+D2), 1.85 (m, 1H, CH Ile, D1), 1.91 (m, 1H, CH Ile, D2), 1.98 (br, 3H, H-3, D1+D2), 3.69 (m, 1H, H-3′, D1+D2), 3.81 (br t, 1H, CH-Ile, D1), 3.87 (br t, 1H, CH-Ile, D2), 5.06 (br, 1H, NH, D1), 5.13 (br, 1H, NH, D2), 5.80 (br, 1H, NH, H-4′, D1), 5.87(br, 1H, NH, H-4′ D2); **^{13}C-NMR:** ($CDCl_3$) δ (ppm): 11.5 (CH_3, Ile, D1), 11.2 (CH_3, Ile, D2) 14.6 (CH_3, C-2′, D1+D2), 15.6 (CH_3, Ile, D1), 15.9 (CH_3, Ile, D2), 24.6 (CH_2 Ile, D1), 24.9 (CH_2 Ile, D2), 28.3 (CH_3, C-3*), 28.3 ($C(CH_3)3$*), 35.7 (C-1), 36.7(CH, Ile, D1), 36.8(CH, Ile, D2),), 37.0 (CH_2, C-4, D1+D2), 38.4 (CH_2, C-2, D1+D2), 53.1 (CH, C-1′, D1+D2), 59.7 (CH, Ile, D1), 59.9 (CH, Ile, D2), 79.8 (Cq, tBu, D1), 79.9(Cq, tBu, D2), 155.8 (CO-Boc, D1+D2), 170.9 (CO, D1), 170.9 (CO, D2); **ESI-MS:** 585 $[2M+H]^+$, 293 $[M+H]^+$; **ESI-MS:** 807 $[2M+Na]^+$, 415 $[M+Na]^+$; m.p.=152-154 °C; **yield = 62%.**

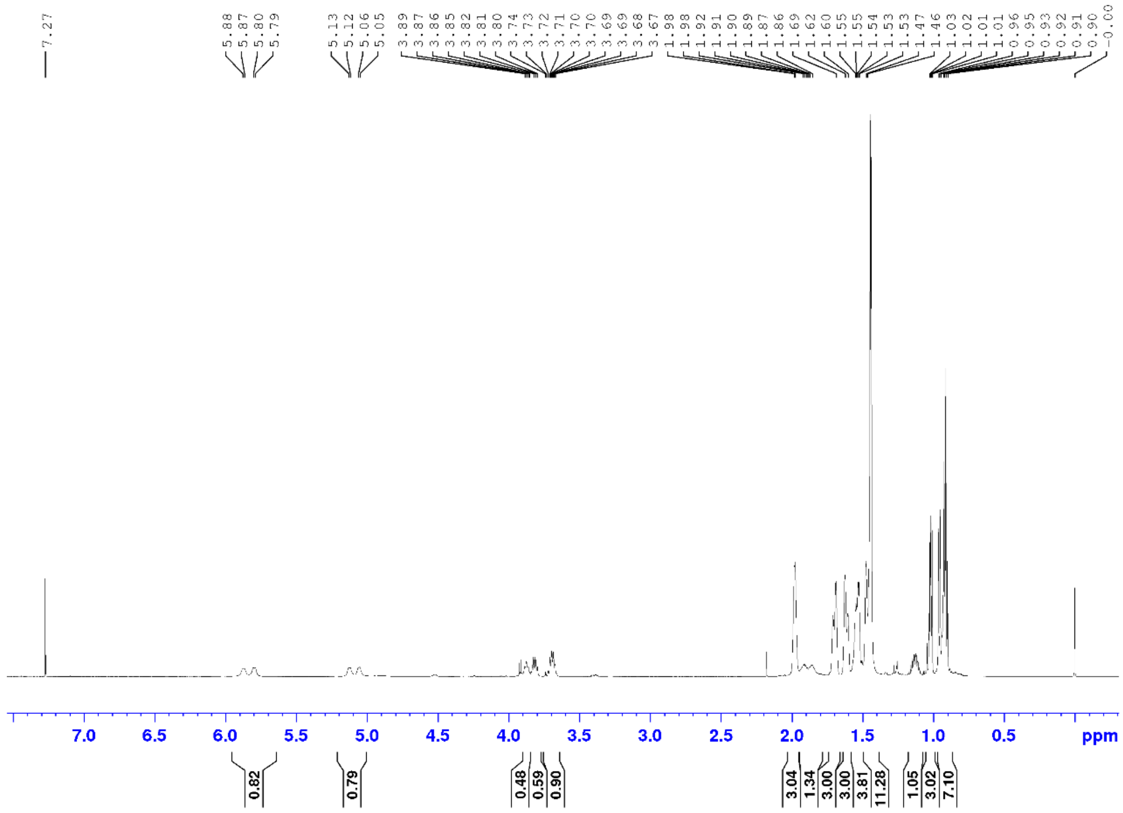

Figure S5. 1D ^{1}H NMR spectrum Isoleucyl-rimantadine

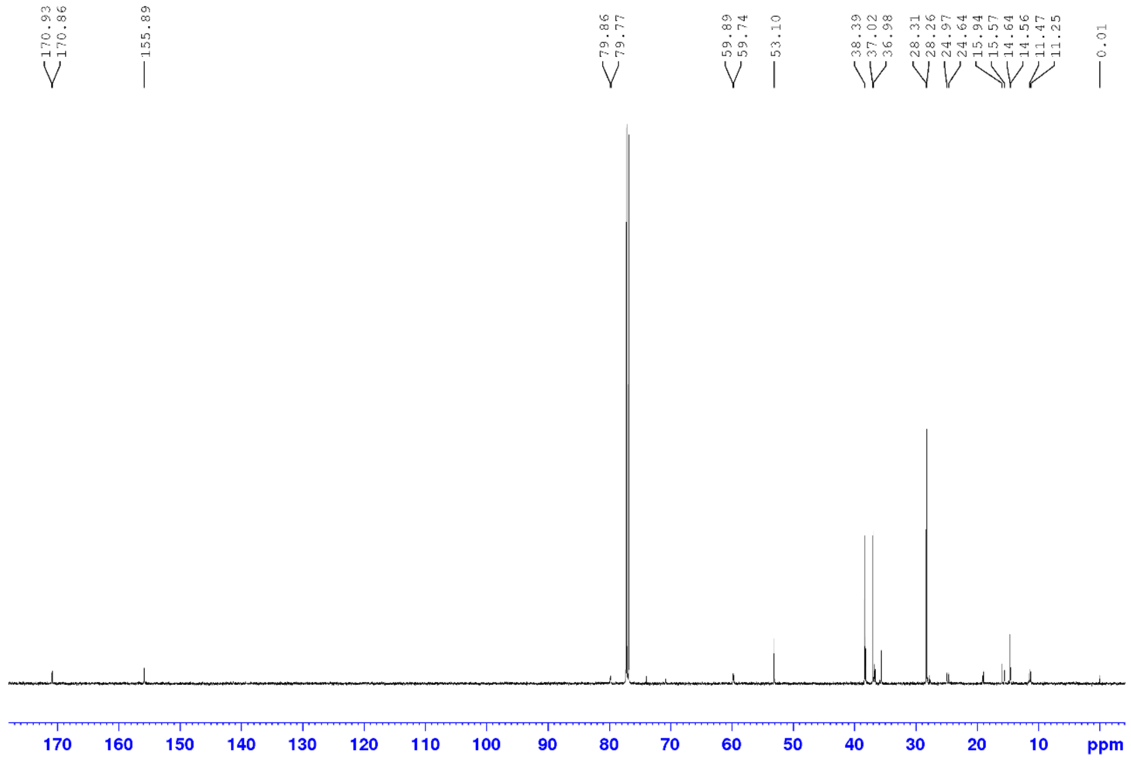

Figure S6. 1D ^{13}C NMR spectra Isoleucyl-rimantadine

Phenylalanyl-rimantadine (4e). **^{1}H-NMR:** ($CDCl_3$) δ (ppm): 0.78(br, 3H, H-2′, D1), 0.935 (d, J=6.9 Hz, 3H, H-2′, D2), 1.283 (m, 6H, H-2a+H-2b, D2), 1.33 (m, 3H, H-2a, D1), 1.41 (m, 3H, H-2b, D1), 1.426 (s, 9H, tBu, D1+D2), 1.523 (m, 3H, H-4b, D2), 1.560 (m, 3H, H-4b,D1), 1.64 (m, 3H, H-4a, D2), 1.66 (m, 3H, H-4a, D1), 2.99 (dd, 1H, 3J=9.6, 2J=13.5, CH_2, PheAla, D1), 3.02 (dd, 1H, 3J=7.9, 2J=13.8, CH_2, PheAla D2), 3.11 (dd, 1H, 3J=6.2, 2J=13.8, CH_2, PheAla, D1), 3.14 (dd, 1H, 3J=5.9, 2J=13.8, CH_2, PheAla, D2), 3.59 (m, 1H, H-3′, D1+D2), 4.24 (br q, 1H, CH, PheAla, D1), 4.30 (br q, 1H, CH, PheAla, D2), 5.03 (br, 1H, NH, D2), 5.20 (br, 1H, NH, D1), 5.41 (br, 1H, NH, D1), 5.73 (br, 1H, NH, D2), 7.23 (m, 3H, D1+D2 o+p-Ar), 7.30 (t, 2H, D1+D2(m-Ar)); **^{13}C-NMR:** ($CDCl_3$) δ (ppm): 14.2 (CH_3, C-2′, D1), 14.4 (CH_3, C-2′, D2), 28.2* (Rim C-3, D2), 28.2** (Rim C-3, D1), 28.3* (C(CH_3)3, D2), 28.3** (C(CH_3)3, D1), 35.4 (Cq, C-1, D2), 35.6 (Cq, C-1, D1), 36.9 (CH_2, C-4, D2), 36.9 (CH_2, C-4, D1), 38.0 (CH_2, D2), 38.1 (CH_2, C-2, D1), 38.2 (Rim-CH_2, C-2, D1), 38.2 (PheAla-CH_2, D1), 38.6 (PheAla-CH_2, D2), 53.0 (CH, C-1′, D1), 53.1(CH, C-1′, D2), 56.2 (Rim-CH, D2+D1), 80.1 (Cq, tBu, D1+D2), 126.9 (Ph, D1+D2), 128.7 (Ph, D1+D2), 129.4 (Ph, D1+D2), 136.7 (Cq-Ar, D1+D2), 155.4 (CO-Boc,D1+D2), 170.2 (CO, D1), 170.3 (CO, D2); **ESI-MS:** 465 $[M+K]^+$, 449 $[M+Na]^+$, 427 $[M+H]^+$; m.p.=209-211 °C; **yield = 38%.**

•

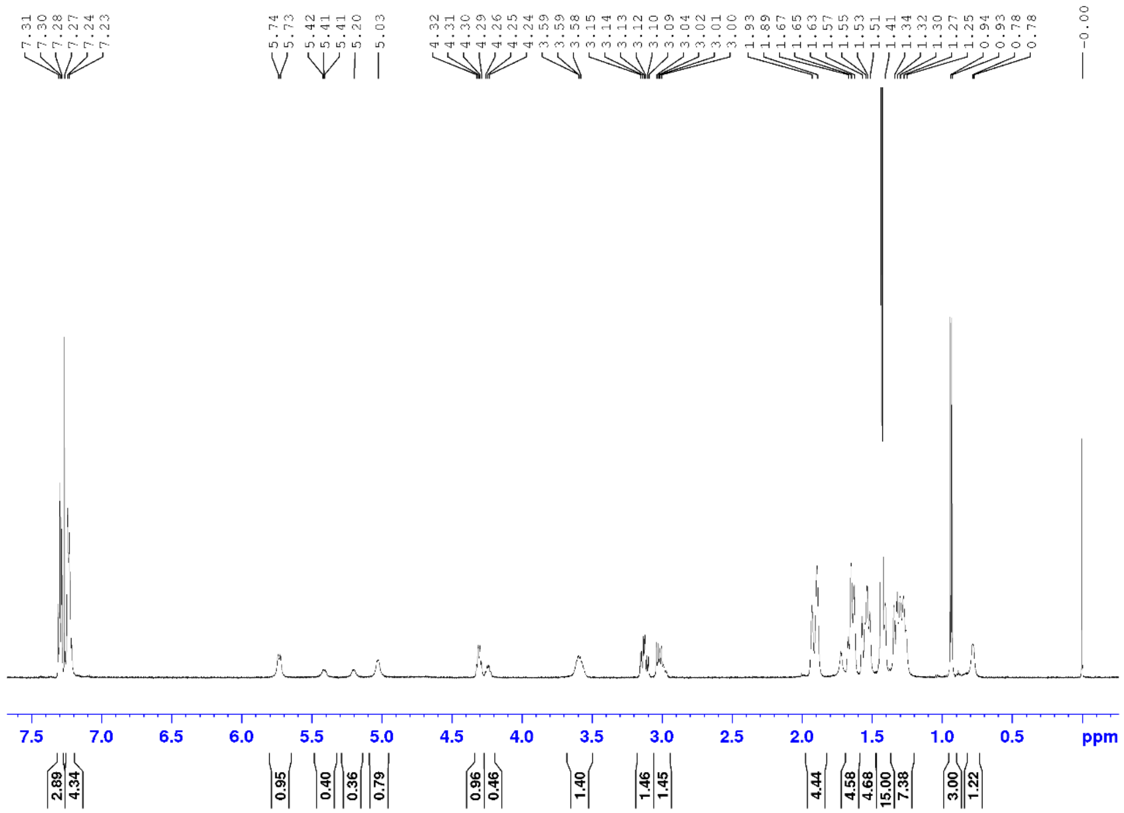

Figure S7. 1D ¹H NMR spectrum Phenylalanyl-rimantadine

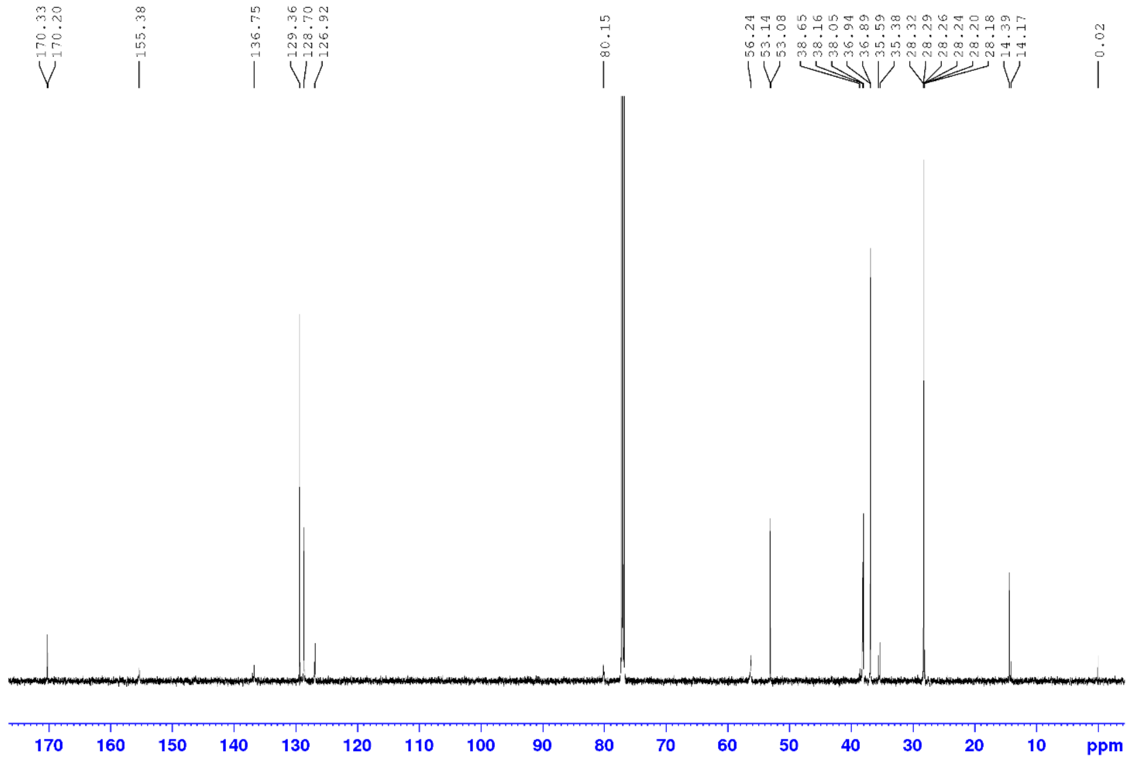

Figure S8. 1D ^{13}C NMR spectra Phenylalanyl-rimantadine

D-(4-F)-phenylalanyl-rimantadine (4f). **^{1}H-NMR:** ($CDCl_3$) δ (ppm): 0.824 (br, 3H, H-2′, D2), 0.946 (d, J=6.9 Hz, 3H, H-2′, D1), 1.290 (br, 6H, H-2a+H2b, D1), 1.363 (m, 3H, H-2a, D2), 1.429 (s, 9H, tBu, D1+D2), 1.430 (m, 3H, H-2b, D2), 1.523 (m, 3H, H-4b, D1), 1.570 (m, 3H, H-4b, D2), 1.653 (m, 3H, H-4a,D1), 1.670 (m, 3H, H-4a, D2), 1.905 (br, 3H, H-3, D1), 1.940 (br, 3H, H-3, D2), 3.02 (m, 2H, D2), 3.06 (m, 2H, D1), 3.59 (m, 1H, H-3′, D1+D2), 4.20 (br q, 1H, D2), 4.260 (br q, 1H, D1), 5.02 (br, 1H, NH, D1), 5.15 (br, 1H, NH, D2), 5.53 (br, 1H, NH, D2), 5.76 (br, 1H, NH, D1), 6.980 (t, 2H, D1+D2), 7.20 (dd, 2H, D1+D2).**^{13}C-NMR:** ($CDCl_3$) δ (ppm): 14.3 (CH_3, C-2′, D2), 14.4 (CH_3, C-2′, D1), 28.2* (Rim C-3, D1), 28.2** (Rim C-3, D2), 28.3* ($C(CH_3)3$, D1), 28.3** ($C(CH_3)3$, D2), 35.4 (Cq, C-1, D1), 35.6 (Cq, C-1, D2), 36.9 (CH_2, C-4, D1), 36.9 (CH_2, C-4, D2), 37.4 (CH_2, D1), 37.6 (CH_2, D2), 38.1 (CH_2, C-2, D1), 38.2 (CH_2, C-2, D2), 53.1 (CH, C-1′, D2), 53.2 (CH, C-1′D1), 56.3 (CH, D1+D2), 80.2 (Cq, tBu, D1+D2), 115.5 (d, 2JC-F=21 Hz, D1+D2), 130.9 (d, 3JC-F=8 Hz, D1+D2), 132.7 (D1+D2), 155.4 (CO-Boc, D2), 155.5 (CO-Boc, D1), 161.9 (d, 1JC-F=245 Hz, D1+D2), 170.1 (CO, D2), 170.1 (CO, D1); **ESI-MS:** 483 $[M+K]^+$, 467 $[M+Na]^+$, 445 $[M+H]^+$; m.p.=228-231 °C; **yield = 54%.**

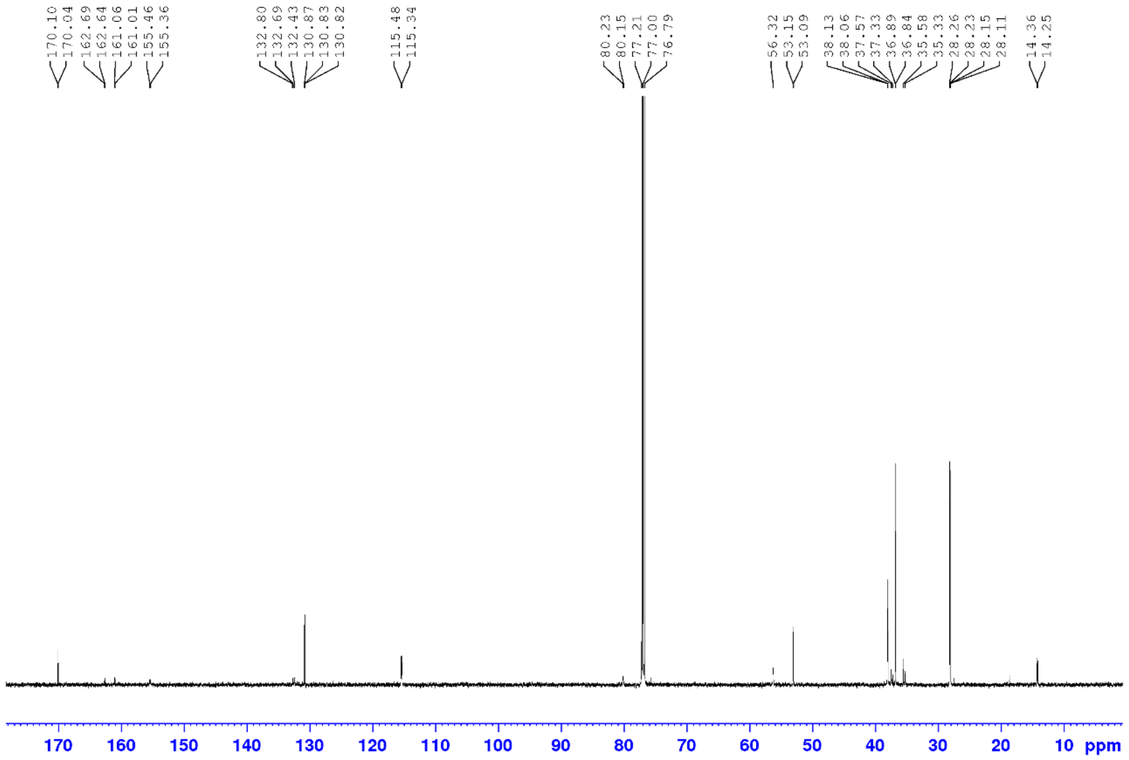

Figure S9. 1D ^{1}H NMR spectrum D-(4-F)-phenylalanyl-rimantadine

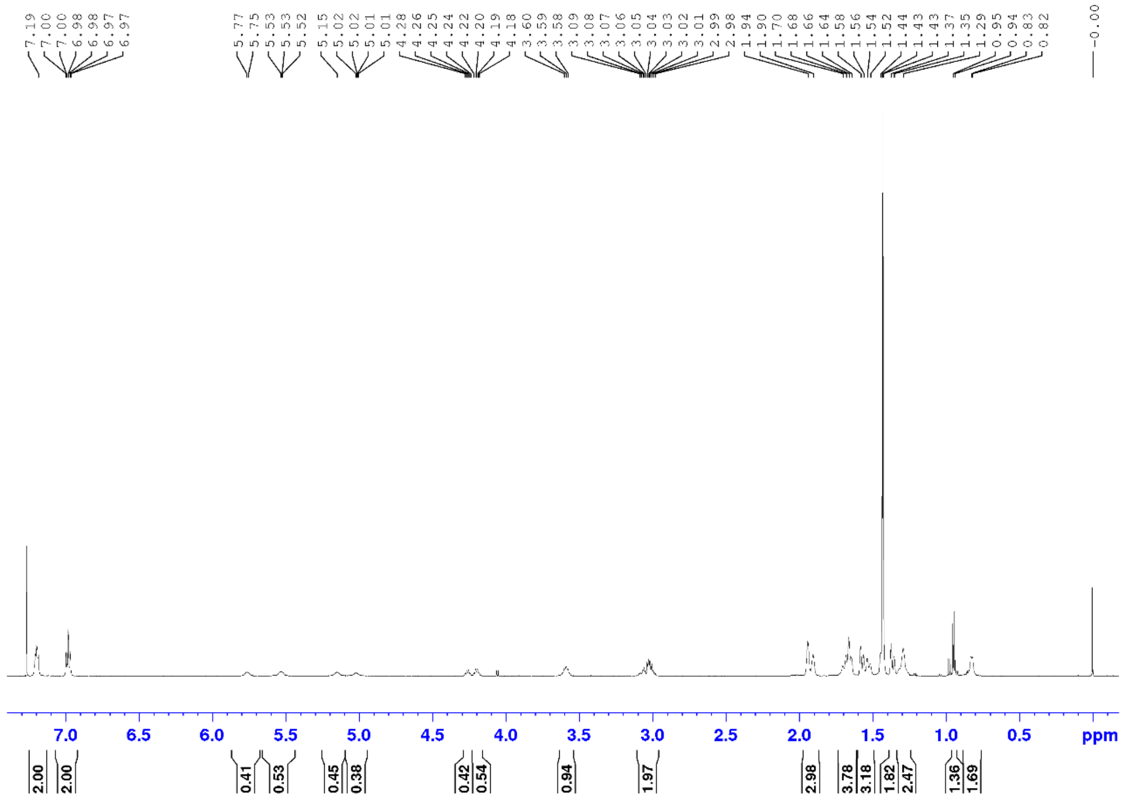

Figure S10. 1D ^{13}C NMR spectra D-(4-F)-phenylalanyl-rimantadine

L-(4-F)-Phenylalanyl-rimantadine (4g). **^{1}H-NMR:** ($CDCl_3$) δ (ppm): 0.822 (br, 3H, H-2′, D2), 0.948 (d, J=6.9 Hz, 3H, H-2′, D1), 1.289 (br, 6H, H-2a+H2b, D1), 1.361 (m, 3H, H-2a, D2), 1.43 (s, 9H, tBu, D1+D2), 1.430 (m, 3H, H-2b, D2), 1.522 (m, 3H, H-4b, D1), 1.571 (m, 3H, H-4b, D2), 1.654 (m, 3H, H-4a, D1), 1.670 (m, 3H, H-4a, D2), 1.905 (br, 3H, H-3, D1), 1.940 (br, 3H, H-3, D2), 3.02 (m, 2H, D2), 3.06 (m, 2H, D1), 3.59 (m, 1H, H-3′, D1+D2), 4.20 (br q, 1H, D2), 4.260 (br q, 1H, D1),5.02 (br, 1H, NH, D1), 5.15 (br, 1H, NH, D2), 5.53 (br, 1H, NH, D2), 5.76 (br, 1H, NH, D1), 6.980 (t, 2H, D1+D2), 7.20 (dd, 2H, D1+D2).**^{13}C-NMR:** ($CDCl_3$) δ (ppm): 14.3 (CH_3, C-2′, D2), 14.4 (CH_3, C-2′, D1), 28.2* (Rim C-3, D1), 28.2** (Rim C-3, D2), 28.3* ($C(CH_3)3$, D1), 28.3** ($C(CH_3)3$, D2), 35.4 (Cq, C-1, D1), 35.6 (Cq, C-1, D2), 36.9 (CH_2, C-4, D1), 36.9 (CH_2, C-4, D2), 37.4 (CH_2, D1), 37.6 (CH_2, D2), 38.1 (CH_2, C-2, D1), 38.2 (CH_2, C-2, D2), 53.1 (CH, C-1′, D2), 53.2 (CH, C-1′D1), 56.3 (CH, D1+D2), 80.2 (Cq, tBu, D1+D2), 115.5 (d, 2JC-F=21 Hz, D1+D2), 130.9 (d, 3JC-F=8 Hz, D1+D2), 132.7 (D1+D2), 155.4 (CO-Boc, D2), 155.5 (CO-Boc, D1), 161.9 (d, 1JC-F=245 Hz, D1+D2), 170.1 (CO, D2), 170.1 (CO, D1); **ESI-MS:** 483 $[M+K]^+$, 467 $[M+Na]^+$, 445 $[M+H]^+$; m.p.=236-238 °C; **yield = 59%.**

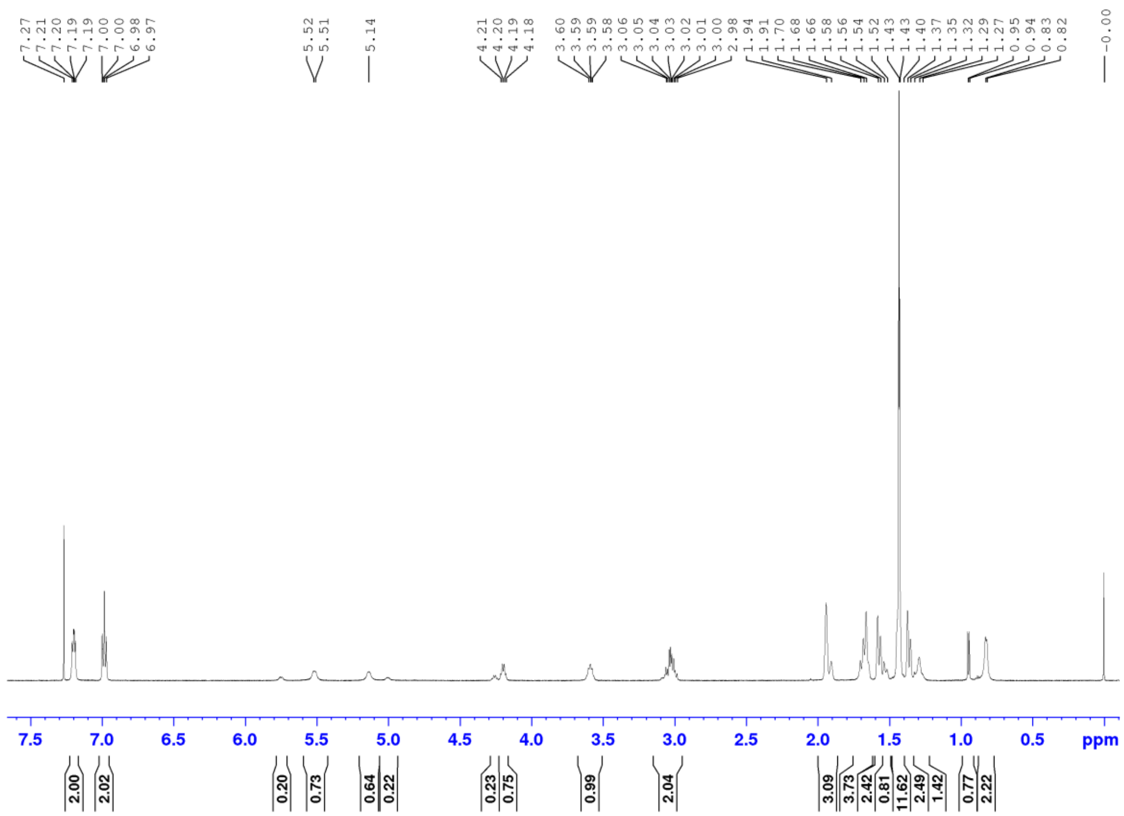

Figure S11. 1D ^{1}H NMR spectrum L-(4-F)-Phenylalanyl-rimantadine

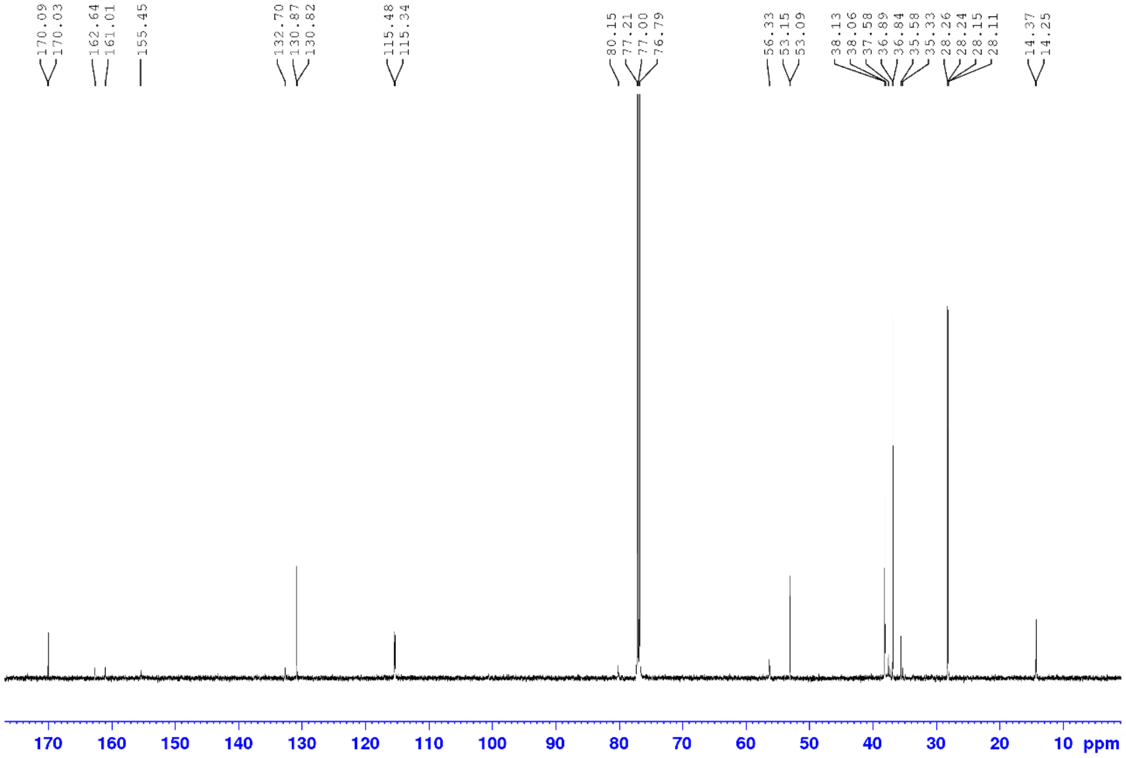

Figure S12. 1D ^{13}C NMR spectra L-(4-F)-Phenylalanyl-rimantadine

Valyl-rimantadine (4h). **^1H-NMR:** ($CDCl_3$) δ (ppm): 0.938 (d, J=7.0 Hz, 3H, CH3-Val), 0.955 (d, J=7.0 Hz, 3H, CH3-Val), 1.023 (d, J=6.9 Hz, 3H, H-2'), 1.447 (s, 9H, tBu), 1.464 (m, 3H, H-2a), 1.536 (m, 3H, H-2b), 1.615 (m, 3H, H-4a), 1.697 (m, 3H, H-4b), 1.976 (br t, 3H, H-3), 2.11 (br, 1H, CH-Val), 3.7 (dq, J=6.9 Hz,1H, CH-valin), 3.77 (br t, J=8 Hz, 1H, RimCH), 5.08 (br d, J=7.8 Hz,1H 1H, NH), 5.73 (br d, J=8.2 Hz, NH); **^{13}C-NMR:** ($CDCl_3$) δ (ppm): 14.7 (CH_3, C-2'), 18.2 (Val-CH_3), 19.4 (Val-CH_3), 28.3 (CH_3)*, 28.3 (C(CH_3)3)*, 30.5 (CH-Val), 35.6 (Cq, C-1), 37.0 (CH_2,C-4), 38.2 (CH_2, C-2), 53.1 (CH-Val), 60.7 (CH, C-1'), 79.8 (Cq, tBu), 155.9 (CO-Boc), 170.8(CO); **ESI-MS:** 417 $[M+K]^+$, 401 $[M+Na]^+$, 379$[M+H]^+$; m.p.=226-228 °C; **yield = 59%.**

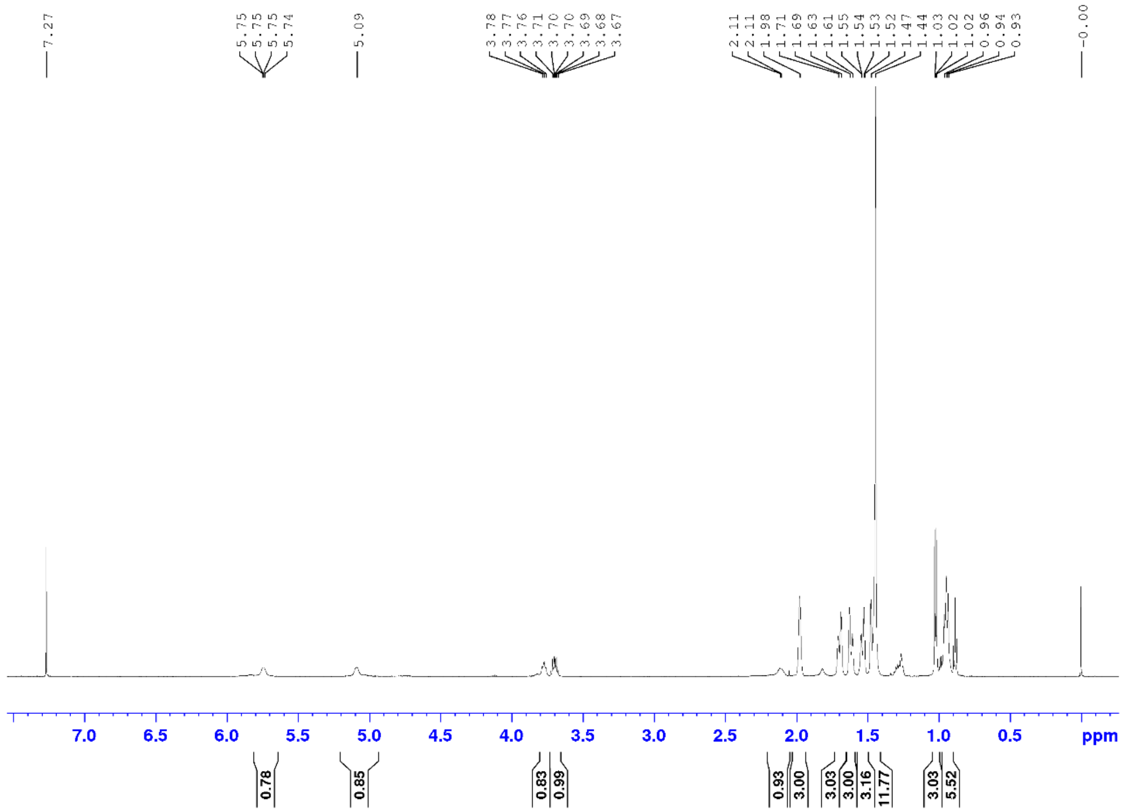

Figure S13. 1D ^{1}H NMR spectrum Valyl-rimantadine

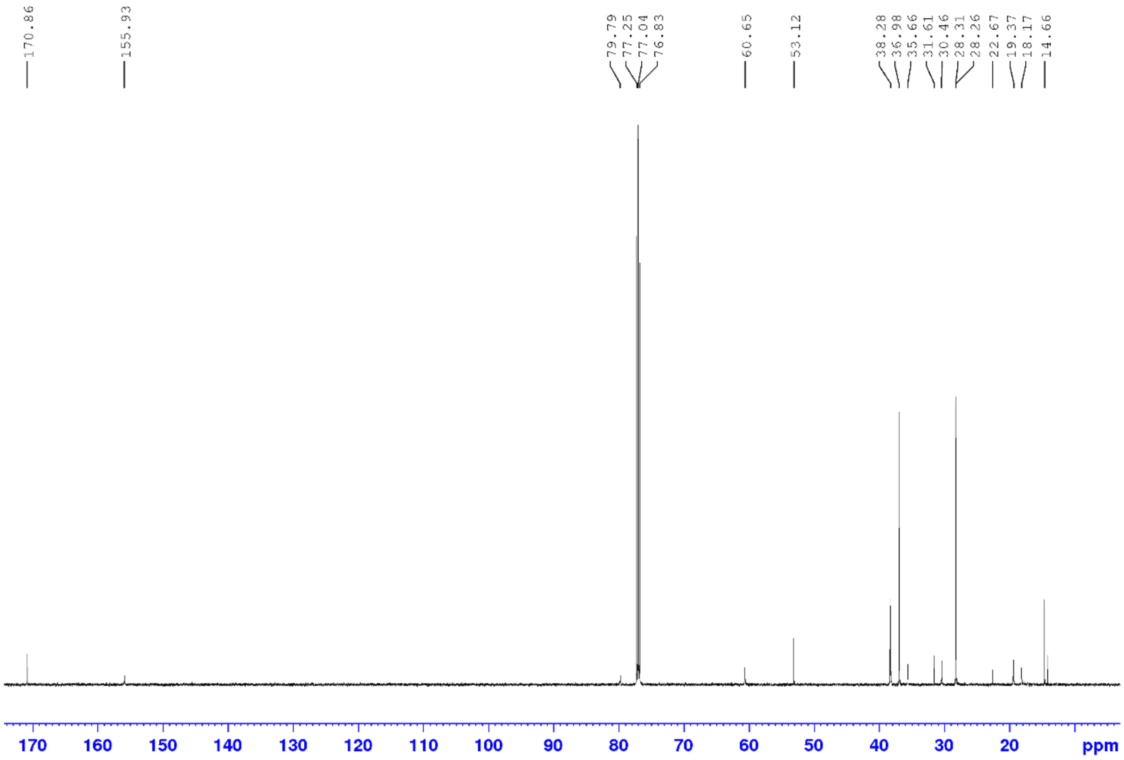

Figure S14. 1D ^{13}C NMR spectra Valyl-rimantadine

β-alanyl-rimantadine (4h). ^{1}H-NMR: ($CDCl_3$) δ (ppm): 1.03. (d, J=5.7 Hz, 3H, H-2′, D1), 1.01. (d, J=5.9 Hz, 3H, H-2′, D2), 2.54. (d, J=6.8 Hz, 3H, CH_2-βAla, D1), 2.57. (d, J=6.8 Hz, 3H, CH_2-βAla, D2), 1.458 (s, 9H, tBu, D1+D2), 1.465 (m, 3H, H-2a*, D1+D2), 1.522 (m, 3H, H-2b*, D1+D2), 1.611 (m, 3H, H-4b, D1+D2), 1.708 (m, 3H, H-4a, D1+D2), 1.986 (br t, 3H, H-3, D1+D2), 3.659 (br, 3H, H-3′,D1+D2), 3.170 (br, 1H, CH_2-βAla, D1+D2), 4.97 (br, 1H, NH), 6.106 (br, 1H, NH, D1), 6.04(br, 1H, NH, D2); **ESI-MS:** 389 $[M+K]^+$, 373 $[M+Na]^+$, 351 $[M+H]^+$; m.p.=164-168 °C; **yield = 70%.**

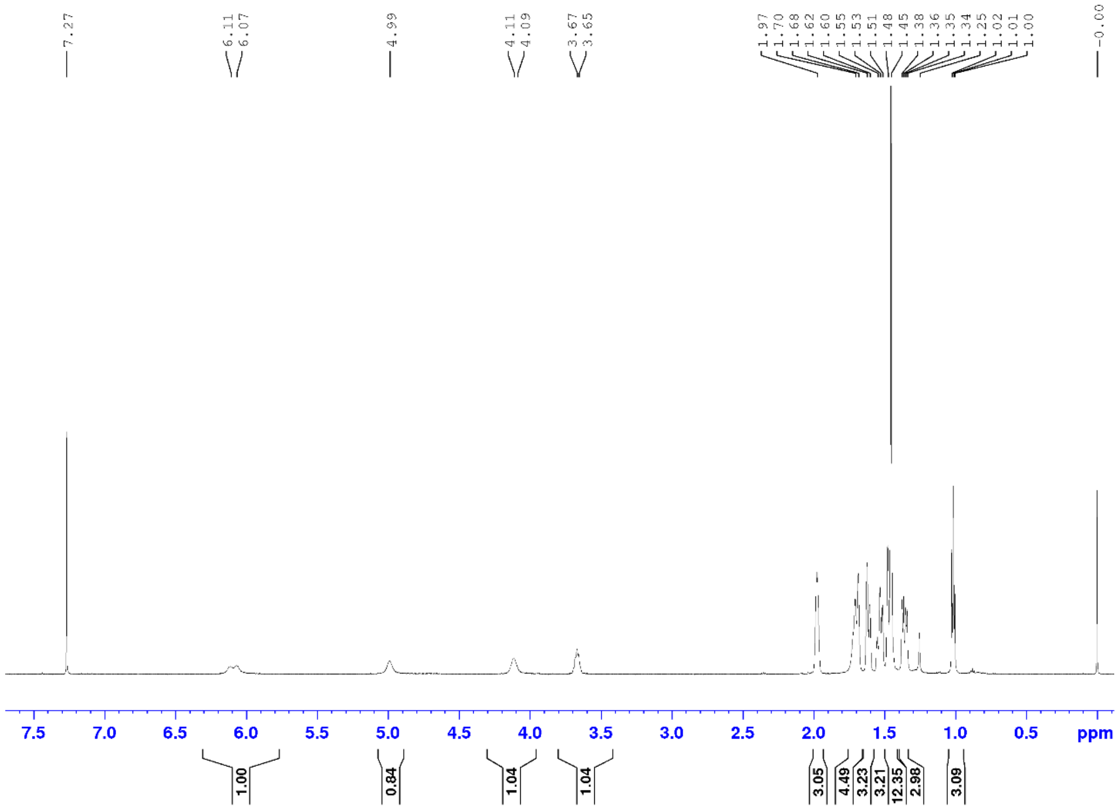

Figure S15. 1D ^{1}H NMR spectrum Alanyl-rimantadine

Tyrosinyl-rimantadine (4j). ^{1}H-NMR: (DMSO-d6) δ (ppm): 0.75. (d, J=6.7 Hz, 3H, H-2′, D2), 0.93 (d, J=6.7 Hz, 3H, H-2′, D1), 1,18 (3H, H-2′, D1), 1,26 (3H, H-2′, D1), 1,45 (6H, H-2′, D2), 1,49 (3H, H-4b′, D1), 1,57 (3H, H-4a′, D1). 1,57 (3H, H-4b′, D2), 1,65 (3H, H-4a′, D2), 1.84 (br, 3H, H-3, D1), 1.93 (br, 3H, H-3, D2), 2.87 (m, 2H, CH_2, D1+D2), 3.44 (D1+D2) 3.93 (m, 1H, CH, D2), 3.99 (m, 1H, CH, D1), 6.69 (m, 2H, Ar, D1), 6.70 (m, 2H, Ar, D2), 7.04 (m, 2H, Ar, D1), 7.01 (m, 2H, Ar, D2), 7.78 (d, 1H, J=9.4 Hz, NH - amide, D2), 7.89 (d, 2H, J=9.4 Hz, NH - amide, D1), 8.12 (br., 1H, NH_2, D2), 8.17 (br, 2H, NH_2, D1); **^{13}C-NMR:** (DMSO-d6) δ (ppm): 13.6 (CH_3, D2), 13.9 (CH_3, D1), 27.5 (CH, rimantadine, D1), 27.6 (CH, rimantadine, D2), 36.3 (CH_2, D1+D2), 37.5 (C-4, rimantadine, D1), 37.6 (C-4, rimantadine, D2), 37.5 (C-2, rimantadine, D1), 37.6 (C-2, rimantadine, D2), 52.7 (CH, D1), 52.6 (CH, D1), 53.6 (CH, D1), 53.5 (CH, D1), CQaurimantadine. 115 (CH-Ar, D1+D2), 124,7 (Cquat, D1+D2) 130.4 (CH-Ar, D2) 130.2 (CH-Ar, D2), 156.5 (Cquat, D1), 156.4 (Cquat, D2), 167.1 (CO, D1), 167.1 (CO, D2); **ESI-MS:** 537 $[M+K]^+$, 521 $[M+Na]^+$, 499$[M+H]^+$; m.p.=185-186 °C; **yield = 56%.**

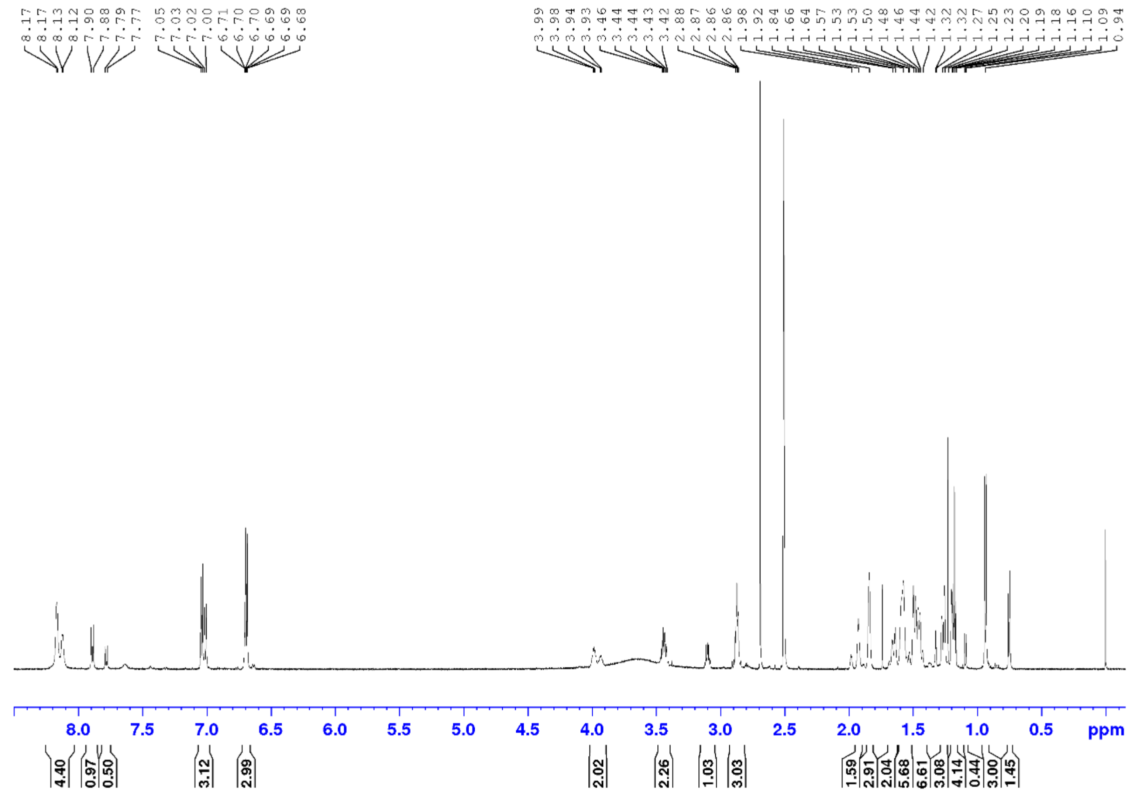

Figure S16. 1D ^{1}H NMR spectrum Tyrosinyl-rimantadine

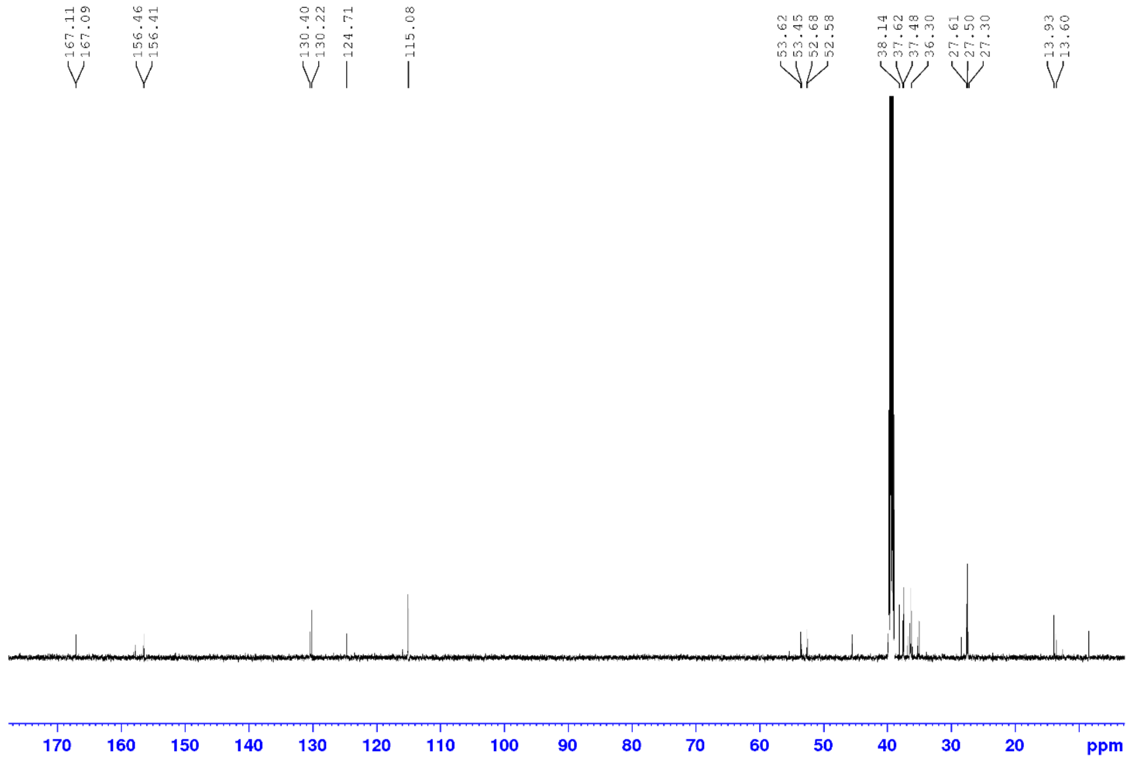

Figure S17. 1D ^{13}C NMR spectra Tyrosinyl-rimantadine

Alanyl-amantadine (5a). ^{1}H-NMR: ($CDCl_3$) δ (ppm): 1.30, (d, J=7Hz, 3H), 1.44 (s, 9H), 1.66 (s, 6H), 1.973 (s, 6H), 2.065 (s, 3H), 4.024 (br s, 1H, CH), 5.024 (br s, 1H, NH), 5.796 (br s, 1H, NH); **^{13}C-NMR**: ($CDCl_3$) δ (ppm): 18.3 (CH_3), 28.3 ($C(CH_3)3$), 29.4 (3xCHam), 36.3 (CH_2), 41.5 (CH_2), 50.5 (CH), 51.9 (Cq-Am), 80.0 ($C(CH_3)3$) 155.8, 171.61; **ESI-MS:** 361 $[M+K]^+$, 345 $[M+Na]^+$, 323 $[M+H]^+$; m.p.=255-257 °C;**yield = 56%.**

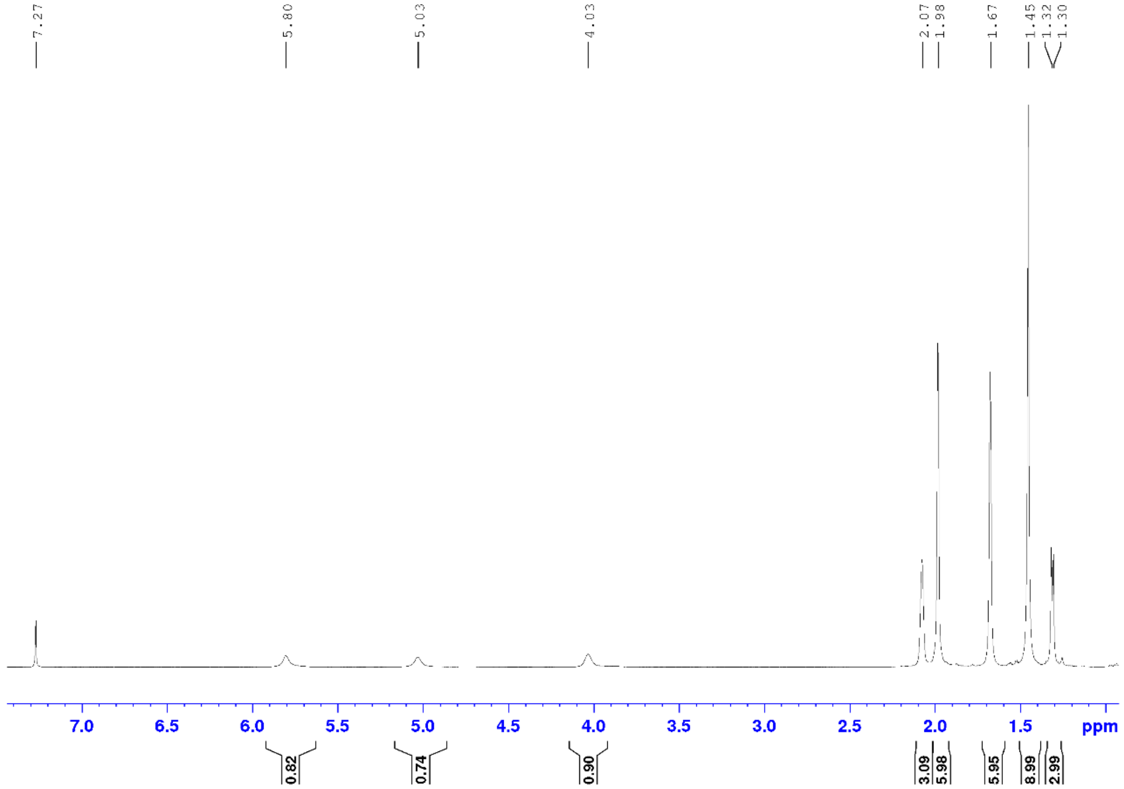

Figure S18. 1D ^{1}H NMR spectrum Alanyl-amantadine

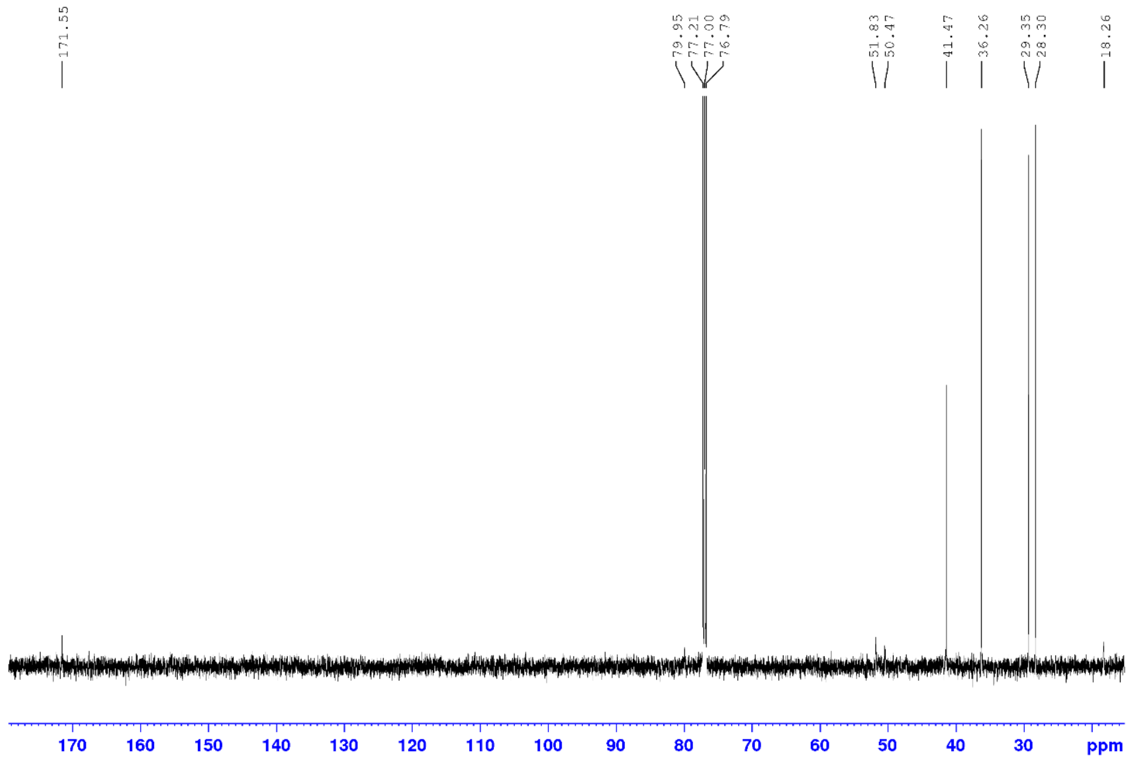

Figure S19. 1D ^{13}C NMR spectra Alanyl-amantadine

Phenylalanyl-amantadine (5e). ^{1}H-NMR: (DMSO-d6) δ (ppm): 1.57. (d, J=12,8 Hz, 3H, amantadine), 1.61 (d, J=12,8 Hz, 3H, amantadine), 1.83 (s, 6H, amantadine), 1.99 (s, 3H, amantadine), 2.87 (dd, , J=13,8 Hz, J=7,1 Hz,1H, CH_2), 2.94 (dd, , J=13,4 Hz, J=7,1 Hz,1H, CH_2), 3.76 (t, 1H, J=7.1 Hz, CH), 7.23 (m, 2H, o-Ph), 7.26 (m, 1H, p-Ph), 7.32 (m, 2H, m-Ph); **^{13}C-NMR:** (DMSO-d6) δ (ppm): 28.6 (CH, amantadine), 35.7 (CH_2, amantadine), 38.1 (CH_2), 40.6(CH_2, amantadine), 50.9 (Cquat, amantadine), 54.0 (CH), 126.7 (CH-Ar) 128.2 (CH-Ar) 129.4 (CH-Ar)135.7 (Cquat), 168.1 (CO); ESI-MS: 437 $[M+K]^+$, 421 $[M+Na]^+$, 399 $[M+H]^+$; m.p.=254-256 °C;**yield = 44%.**

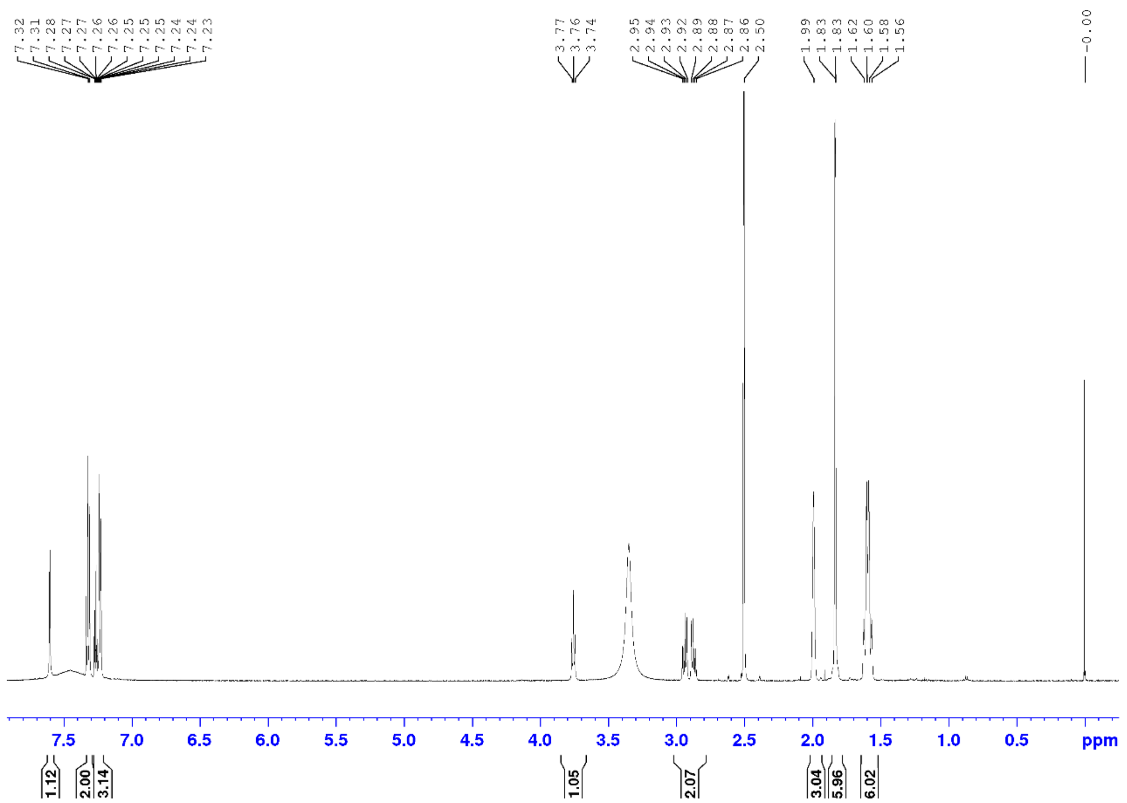

Figure S20. 1D ^{1}H NMR spectrum Phenylalanyl-amantadine

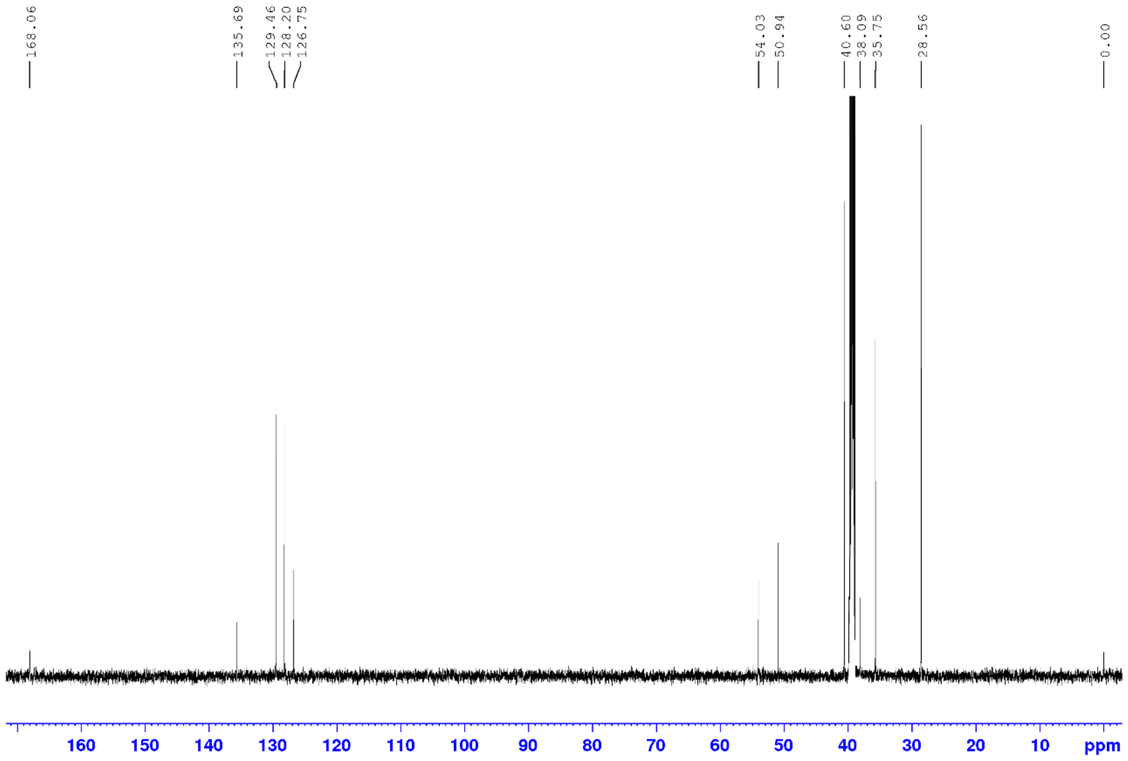

Figure S21. 1D ^{13}C NMR spectra Phenylalanyl-amantadine

(4-F)-Phenylalanyl-amantadine (5j). **^{1}H-NMR:** (DMSO-d6) δ (ppm): 1.58, (d, J=12,8 Hz, 3H, amantadine), 1.621 (d, J=12,8 Hz, 3H, amantadine), 1.85 (s, 6H, amantadine), 2.00 (s, 3H, amantadine), 2.88 (dd, , J=13,8 Hz, J=7,1 Hz,1H, CH_2), 2.95 (dd, , J=13,8 Hz, J=6,9 Hz,1H, CH_2), 3.77 (t, 1H, J=7.1 Hz, CH), 7.16 (m, 2H, o-Ph), 7.26 (m, 1H, p); **^{13}C-NMR:** (DMSO-d6) δ (ppm): 28.6 (CH, amantadine), 35.7 (CH_2, amantadine), 37.0 (CH2), 40.6(CH_2, amantadine), 51.0 (Cquat, amantadine), 53.9 (CH), 115.0 (CH-Ar) 131.4 (CH-Ar) 131.6 (Cquat)161.3 (Cquat), 168.6 (CO); **ESI-MS:** 455 $[M+K]^+$, 439 $[M+Na]^+$, 417 $[M+H]^+$; m.p.=247-249 °C; **yield = 55%.**

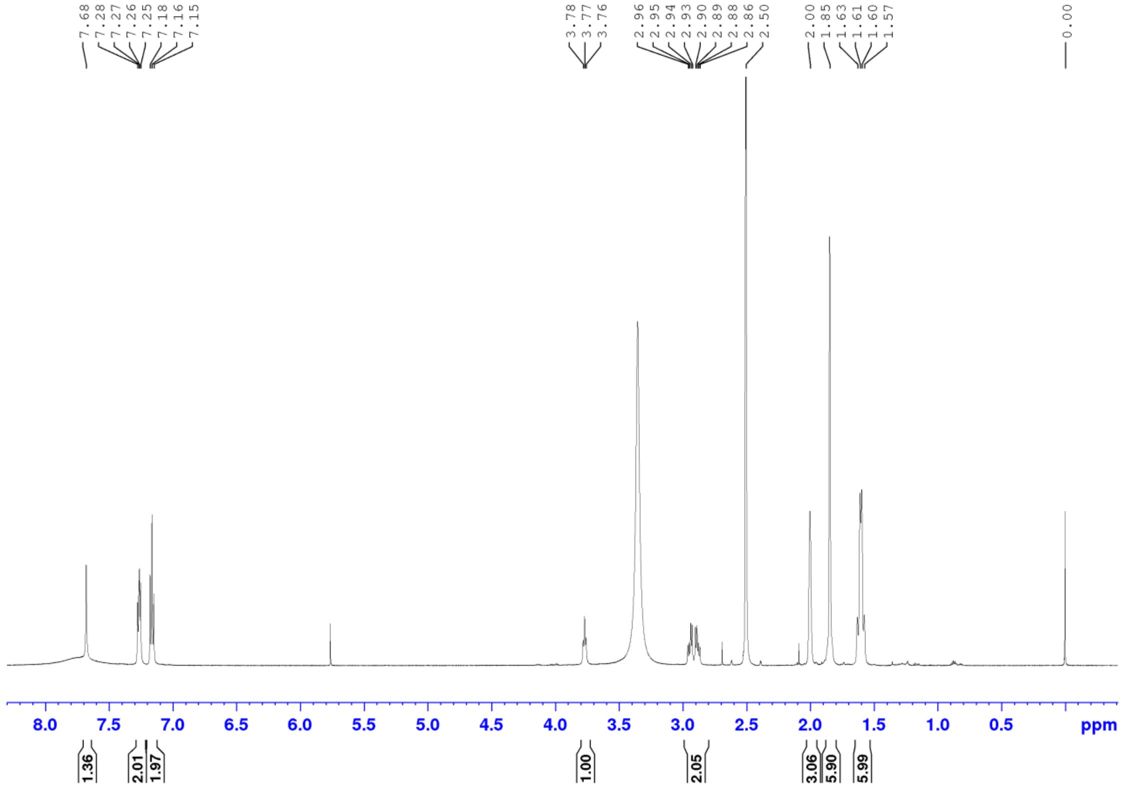

Figure S22. 1D ^{1}H NMR spectrum (4-F)-Phenylalanyl-amantadine

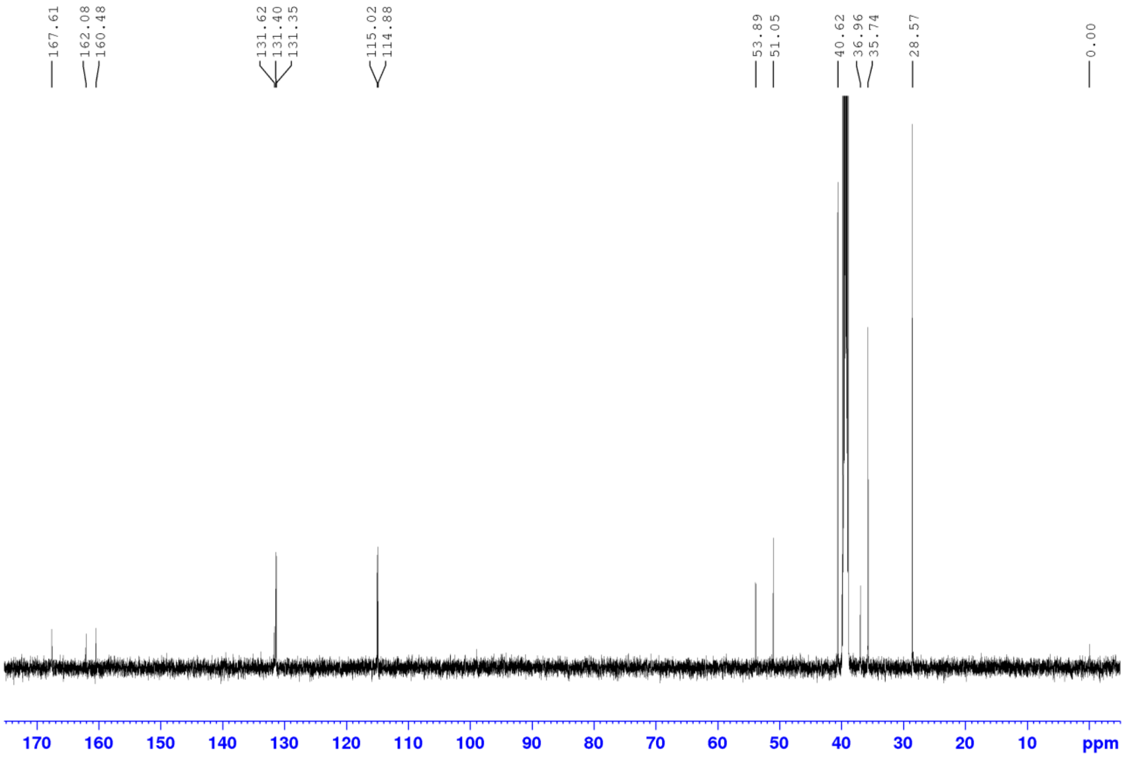

Figure S23. 1D ^{13}C NMR spectra (4-F)-Phenylalanyl-amantadine

Valyl-amantadine (5h). ^{1}H-NMR: ($CDCl_3$) δ (ppm): 0.901 (d, J=6.8 Hz, 3H), 0.937 (d, J=6.5 Hz, 3H), 1.435 (s, 9H), 1.664 (br t, 6H), 1.985 (s, 6H), 2.03 (overlapping, 1H), 2.06 (s, 3H), 3.705 (br t, J= 7.9 Hz, 1H), 5.09 (d, J= 8.2 Hz, 1H), 5.478 (s, 1H); **^{13}C-NMR:** ($CDCl_3$) δ (ppm): 18.0 (CH_3), 19.3 (CH_3), 28.3 ($C(CH_3)3$), 29.4 (3xCHam), 31.2 (CH, valline), 36.3 (CH_2), 41.6 (CH_2), 52.1 (Cq-Am), 60.47 (CH, valline), 79.7 ($C(CH_3)3$), 155.9, 170.4; **ESI-MS:** 389 $[M+K]^+$, 373 $[M+Na]^+$, 351 $[M+H]^+$; m.p.=132-134 °C; **yield = 36%.**

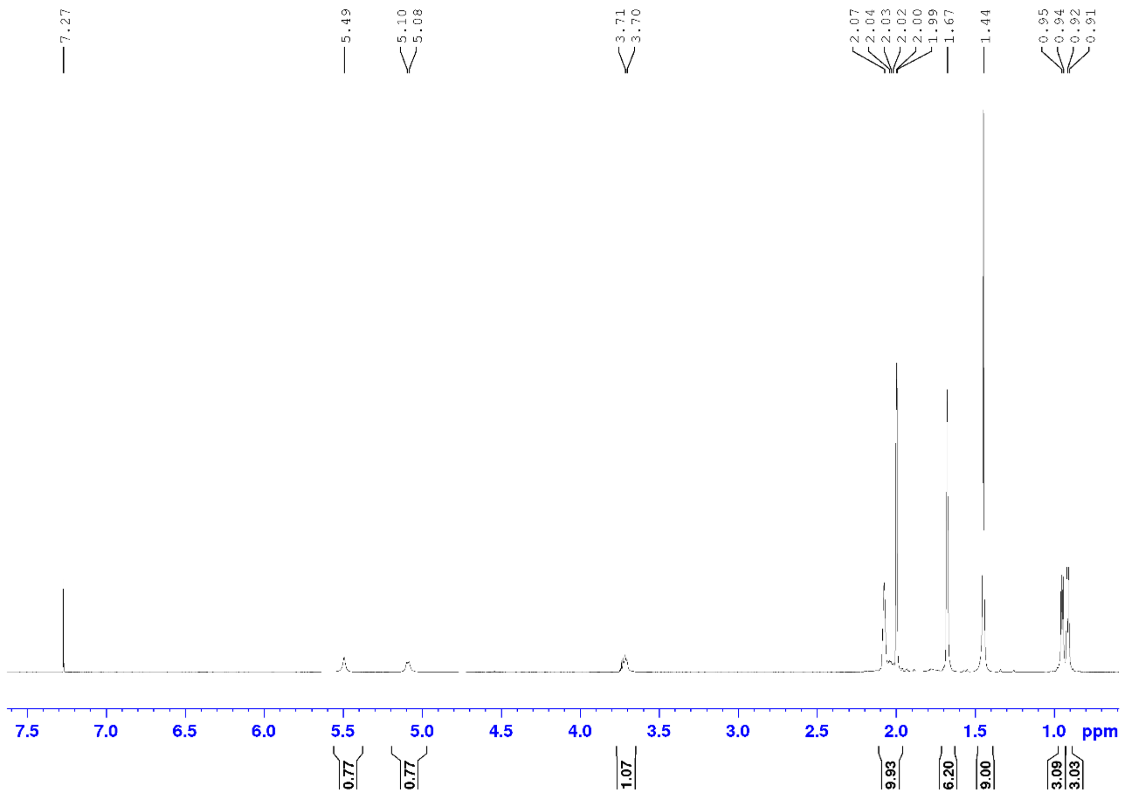

Figure S24. 1D ^{1}H NMR spectrum Valyl-amantadine.

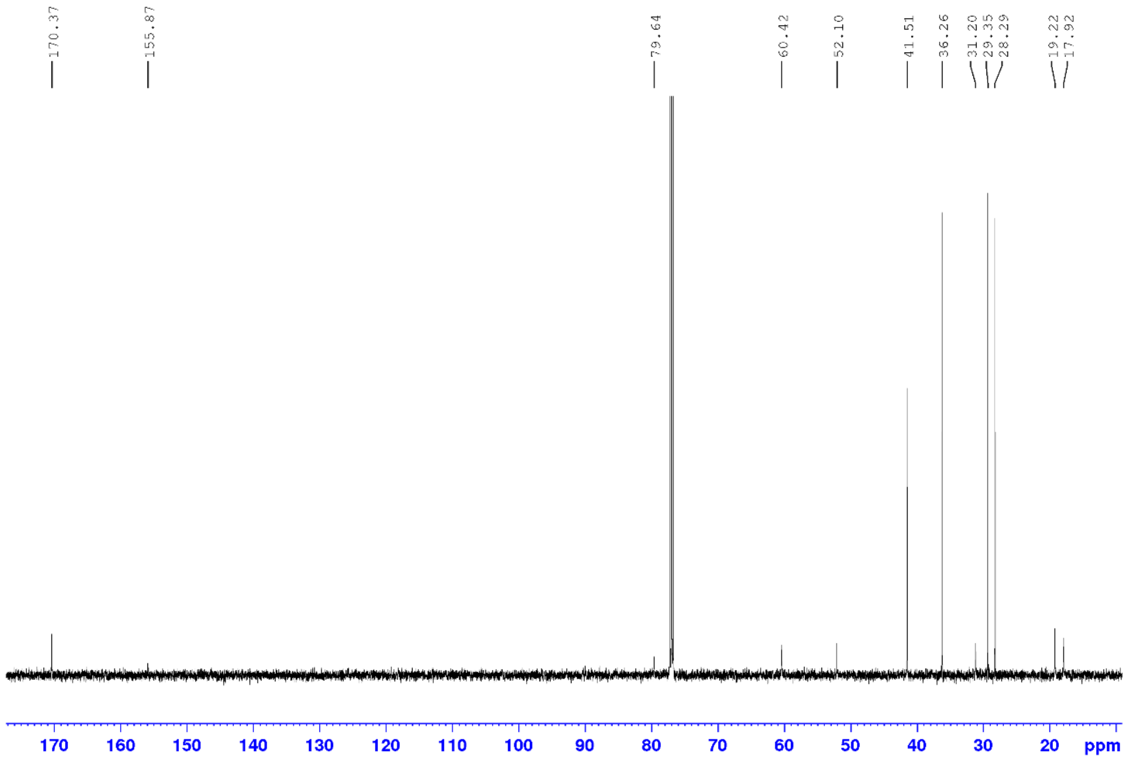

Figure S25. 1D ^{13}C NMR spectra Valyl-amantadine.

Guanidino-alanyl-rimantadine (6a). ^{1}H-NMR: (DMSO-d6) δ (ppm): 1.02. (d, 3H), 1.13 (s, NH), 1.14 (br d, J=10 Hz, 2H), 1.38-1.63 (m, 12), 1.78 (br s, 1H, 1-H), 2.33 (s, 3H, NCH_3), 2.65 (m, 1H, CHN) 3.99 (m, 3H, CH_2CH_3, CHN), 4.28 (br d, 1H, NH), 2.55 (d, J=6.8 Hz, 3H, CH_2-βAla, D2), 3.070 (br, 1H, CH_2-βAla), 5.07 (br, 1H, NH); **ESI-MS:** 531 [M+K]$^+$, 515 [M+Na]$^+$, 493 [M+H]$^+$; m.p.=136-138 °C **;yield = 46%.**

Guanidino-β-alanyl-rimantadine (6i). ^{1}H-NMR: (DMSO-d6) δ (ppm): 1.02. (d, 3H), 1.13 (s, NH), 1.14 (br d, J=10 Hz, 2H), 1.38-1.63 (m, 12), 1.78 (br s, 1H, 1-H), 2.33 (s, 3H, NCH_3), 2.65 (m, 1H, CHN) 3.99 (m, 3H, CH_2CH_3, CHN), 4.28 (br d, 1H, NH), 2.55 (d, J=6.8 Hz, 3H, CH_2-βAla, D2), 3.070 (br, 1H, CH_2-βAla), 5.07 (br, 1H, NH); **ESI-MS:** 585 [2M+H]$^+$, 293 [M+H]$^+$; m.p.=145-147 °C; **yield = 64%.**

Guanidino-tyrosinyl-rimantadine (6j). ^{1}H-NMR: (DMSO-d6) δ (ppm): 0.95. (d, 3H), 1.10 (s, NH), 1.26 (br d, J=10 Hz, 2H), 1.40-1.75 (m, 12), 1.89 (br s, 1H, 1-H), 2.35 (s, 3H, NCH_3), 2.63-2.77(m, 1H, CHN) 3.92-4.21 (m, 3H, CH_2CH_3, CHN), 4.32 (br d, 1H, NH), 6.69 (m, 2H, Ar, D1), 6.70 (m, 2H, Ar, D2), 7.04 (m, 2H, Ar, D1), 7.01 (m, 2H, Ar, D2), 7.78 (d, 1H, J=9.4 Hz, NH - amide, D2), 7.89 (d, 2H, J=9.4 Hz, NH - amide, D1), 8.12 (br., 1H, NH_2, D2), 8.17 (br, 2H, NH_2, D1); **ESI-MS:** 769 [2M+H]$^+$, 385 [M+H]$^+$; m.p.=229-231 °C; **yield = 46%.**

Guanidino-alanyl-amantadine (7a). ^{1}H-NMR: (DMSO-d6) δ (ppm): 1.37 (d, 3H), 1.46 (d, 3H), 1.68 (m, 6H), 2.01(, 3H), 7.86 (s, 3H), 3.708 (br t, J= 7.9 Hz, 1H), 5.05 (d, J= 8.2 Hz, 1H), 5.478 (s, 1H); **ESI-MS:** 529 [2M+H]$^+$, 265 [M+H]$^+$; m.p.=231-233 °C; **yield = 26%.**

Guanidino-valyl-amantadine (7h). ^{1}H-NMR: (DMSO-d6) δ (ppm): 1.57 (d, 3H), 1.66 (d, 3H), 1.78 (m, 6H), 2.08(, 3H), 7.99 (s, 3H), 3.705 (br t, J= 7.9 Hz, 1H), 5.09 (d, J= 8.2 Hz, 1H), 5.478 (s, 1H); **ESI-MS:** 585 [2M+H]$^+$, 293 [M+H]$^+$; m.p.=119-231 °C; **yield = 56%.**

Table S1. Crystallographic data-collection statistics for glycyl-rimantadine.

Identification code	glycyl-rimantadine (4b)
Empirical formula	C16H27N2O4F3
Formula weight	514.38
Temperature/K	293(2)
Crystal system	orthorhombic
Space group	Pbca
a[Å]	9.0304(3)
b[Å]	10.1550(3)
c[Å]	39.4276(13)
α[°]	90
β[°]	90
γ[°]	90
Volume[Å]3	3615.6(2)
Z	5
ϱcalcg/cm3	1.181
μ/mm-1	0.081
F(000)	1359
Crystal size/mm3	0.3 × 0.2 × 0.15
Radiation	MoKα (λ = 0.71073)
2Θ range for data collection[°]	6.118 to 57.074
Index ranges	-12 ≤ h ≤ 11, -12 ≤ k ≤ 13, -52 ≤ l ≤ 46
Reflections collected	20010
Independent reflections	4025 [Rint = 0.0387, Rsigma = 0.0265]
Data/restraints/parameters	4025/0/363
Goodness-of-fit on F2	1.058
Final R indexes [I>=2σ (I)]	R1 = 0.0522, wR2 = 0.1235
Final R indexes [all data]	R1 = 0.0754, wR2 = 0.1365
Largest diff. peak/hole / e Å-3	0.24/-0.15
CCDC number	1940157

Table S2. Selected geometric parameters for Glycyl-rimantadine (4b). Bond Lengths [Å] and Bond Angles [°].

Bond	Length[Å]	Angle	Angle[˚]

O1 – C13	1.226(2)	C13 – N1 – C11	123.47(18)
N2 – C14	1.476(3)	C7 – C1 – C4	108.11(16)
N1 – C13	1.329(3)	C7 – C1 – C11	112.85(16)
N1 – C11	1.467(3)	C2 – C1 – C7	108.10(16)
C1 – C7	1.536(3)	C2 – C1 – C4	108.22(16)
C1 – C2	1.535(3)	C2 – C1 – C11	109.83(15)
C1 – C4	1.541(3)	C4 – C1 – C11	109.61(16)
C1 – C11	1.546(3)	C6 – C7 – C1	110.68(16)
C7 – C6	1.533(3)	C3 – C2 – C1	110.73(16)
C2 – C3	1.525(3)	O1 – C13 – N1	125.55(19)
C13 – C14	1.515(3)	O1 – C13 – C14	120.17(19)
C4 – C9	1.529(3)	N1 – C13 – C14	114.25(18)
C11 – C12	1.527(3)	C9 – C4 – C1	110.82(17)
C3 – C8	1.530(3)	N1 – C11 – C1	111.72(16)
C3 – C5	1.524(3)	N1 – C11 – C12	108.56(19)
C9 – C8	1.519(3)	C12 – C11 – C1	115.41(18)
C9 – C10	1.532(3)	C2 – C3 – C8	109.70(19)
C10 – C6	1.524(3)	C5 – C3 – C2	109.57(18)
C5 – C6	1.529(3)	N2 – C14 – C13	110.45(18)
